# Virtual Reality Analgesia With Interactive Eye Tracking During Brief Thermal Pain Stimuli: A Randomized Controlled Trial (Crossover Design)

**DOI:** 10.3389/fnhum.2019.00467

**Published:** 2020-01-23

**Authors:** Najood A. Al-Ghamdi, Walter J. Meyer, Barbara Atzori, Wadee Alhalabi, Clayton C. Seibel, David Ullman, Hunter G. Hoffman

**Affiliations:** ^1^Department of Computer Science, Faculty of Computing and Information Technology, King Abdulaziz University, Jeddah, Saudi Arabia; ^2^Shriners Hospitals for Children, Galveston, TX, United States; ^3^Department of Psychiatry, The University of Texas Medical Branch at Galveston, Galveston, TX, United States; ^4^Department of Health Sciences, School of Psychology, University of Florence, Florence, Italy; ^5^Department of Computer Science, College of Engineering, Effat University, Jeddah, Saudi Arabia; ^6^The Virtual Reality Research Group, King Abdulaziz University, Jeddah, Saudi Arabia; ^7^Department of Computer Science, Dar Al-Hekma University, Jeddah, Saudi Arabia; ^8^Virtual Reality Research Center, Human Photonics Lab, University of Washington, Seattle, WA, United States; ^9^Department of Mechanical Engineering, College of Engineering, University of Washington, Seattle, WA, United States

**Keywords:** virtual reality, analgesia, pain, distraction, non-pharmacologic analgesic techniques, opioid analgesia

## Abstract

In light of growing concerns about opioid analgesics, developing new non-pharmacologic pain control techniques has become a high priority. Adjunctive virtual reality can help reduce acute pain during painful medical procedures. However, for some especially painful medical procedures such as burn wound cleaning, clinical researchers recommend that more distracting versions of virtual reality are needed, to further amplify the potency of virtual reality analgesia. The current study with healthy volunteers explores for the first time whether interacting with virtual objects in Virtual Reality (VR) via “hands free” eye-tracking technology integrated into the VR helmet makes VR more effective/powerful than non-interactive/passive VR (no eye-tracking) for reducing pain during brief thermal pain stimuli.

**Method:**

Forty eight healthy volunteers participated in the main study. Using a within-subject design, each participant received one brief thermal pain stimulus during interactive eye tracked virtual reality, and each participant received another thermal pain stimulus during non-interactive VR (treatment order randomized). After each pain stimulus, participants provided subjective 0–10 ratings of cognitive, sensory and affective components of pain, and rated the amount of fun they had during the pain stimulus.

**Results:**

As predicted, interactive eye tracking increased the analgesic effectiveness of immersive virtual reality. Compared to the passive non-interactive VR condition, during the interactive eye tracked VR condition, participants reported significant reductions in worst pain (*p* < 0.001) and pain unpleasantness (*p* < 0.001). Participants reported a significantly stronger illusion of presence (*p* < 0.001), and significantly more fun in VR (*p* < 0.001) during the interactive condition compared to during passive VR. In summary, as predicted by our primary hypothesis, in the current laboratory acute pain analog study with healthy volunteers, increasing the immersiveness of the VR system via interactive eye tracking significantly increased how effectively VR reduced worst pain during a brief thermal pain stimulus. Although attention was not directly measured, the pattern of pain ratings, presence ratings, and fun ratings are consistent with an attentional mechanism for how VR reduces pain. Whether the current results generalize to clinical patient populations is another important topic for future research. Additional research and development is recommended.

## Introduction

Excessive pain during medical procedures is a frequent problem, worldwide, and recovering from a severe burn is often unusually painful. As part of the natural healing process, severe burns exfoliate dead skin cells. To prevent infection and speed up healing, wound care nurses remove the bandages, and wipe/scrub the burn wound with a wet washcloth to remove/clean off the thin layer of sloughed off dead skin cells, and other debris.

Despite giving patients powerful analgesia pain medications shortly before wound care, patients typically remain conscious during burn wound care, pain during non-surgical wound debridement of severe burn wounds is often severe to excruciating, and wound care procedures are repeated frequently. Children with large burns typically have their burn wounds cleaned several days per week, during several weeks of hospitalization (Hoffman et al., 2019).

Psychological factors such as fear, anxiety and depression can increase or amplify how much pain patients subjectively experience during painful medical procedures (Hemington et al., 2017), making pain management even more challenging. What people are thinking about, and where patients direct their attention during medical procedures (Heathcote et al., 2017), expectations of pain, and memories of previous painful procedures can increase pain intensity (Melzack and Wall, 1965; Noel et al., 2012, 2015a,b; Fields, 2018; Fischer et al., 2018).

Opioid analgesics are widely regarded as effective and essential tools for acute pain management such as burn wound care (Malchow and Black, 2008; McIntyre et al., 2016; Ballantyne, 2018) but opioid side effects such as nausea, constipation, urinary retention, drowsiness, and lack of appetite (Mendez-Romero et al., 2018) limit opioid dose levels (Cherny et al., 2001). In addition, there are growing concerns about over-prescription of opioids in the United States and Europe (Krane, 2019; Yaster et al., 2019). There are also growing concerns about the opposite problem of limited availability of opioids in under-developed countries (Vijayan, 2011), recent shortages of medical opioid analgesics in the United States, and possible large reductions in availability in the future, in light of stricter laws and large lawsuits against pharmaceutical companies that sell opioid analgesics. In light of growing problems with opioid analgesics, developing effective new non-pharmacologic pain control treatments has become a national and international priority (Keefe et al., 2012, 2018).

Psychological pain control techniques can help reduce reliance on opioids, and may help compensate for undermedication. There is growing evidence that adjunctive immersive virtual reality distraction can help reduce the suffering of patients during medical procedures with little or no side effects from the VR (Hoffman, 1998; Hoffman et al., 2000, 2011; Garrett et al., 2014; Atzori et al., 2018a, b; Indovina et al., 2018). Virtual reality goggles with eye tracking technology embedded into the goggles, have recently become commercially available, and could potentially help make VR more distracting. However, to date, one important gap in the scientific VR analgesia literature is that there have been no PubMed indexed studies using eye tracked Virtual Reality to treat pain.

In previous clinical studies, virtual reality has typically been used in addition to traditional pain medications to help reduce the pain experienced by patients during painful severe burn wound cleaning sessions (Hoffman et al., 2000, 2004a, 2019; Maani et al., 2011a, b; Kipping et al., 2012; Faber et al., 2013; Jeffs et al., 2014; Khadra et al., 2018; McSherry et al., 2018)^1^. In a military study, while in virtual reality, soldiers with combat-related burn injuries spent less time thinking about their pain, patients reported reductions in worst pain intensity and reductions in the emotional component of pain (pain unpleasantness) during VR, and patients reported having more fun when they went into virtual reality during medical procedures, compared to standard of care pain medications alone (Maani et al., 2008, 2011a,b).

The essence of immersive virtual reality analgesia is the patient’s illusion of going to a different place, the subjective experience of “feeling present” in the computer generated world, as if the virtual world is a place the patient is visiting (Slater et al., 1994; Slater and Wilbur, 1997). Researchers argue that the illusion of “being there” in virtual reality is unusually attention grabbing (Hoffman et al., 2000, 2001, 2003b, 2004a, 2004b, 2004c, 2007; Law et al., 2011). For example, people perform more poorly on a divided attention task while in virtual reality (Hoffman et al., 2003b). The perception of pain requires attentional resources. Researchers (e.g., Hoffman, 1998; Hoffman et al., 2000; Birnie et al., 2017), propose that VR uses up so much attention, that the patient’s brain has less attention available to process incoming nociceptive signals traveling from the burn wound to the brain while the wound is being cleaned.

One clinical research study recently explored the use of virtual reality to help reduce the pain of pediatric patients with large severe burn injuries during burn wound cleaning sessions in the intensive care unit of a regional pediatric burn center (Hoffman et al., 2019). Although patients reported large and significant reductions in pain during burn wound care, the researchers recommended that stronger versions of virtual reality need to be developed, in order to better distract patients experiencing such high levels of pain during burn wound cleaning.

Researchers have described and tested design guidelines for how to make VR more effective. Several analog laboratory thermal pain studies have shown that more immersive VR systems designed to elicit a stronger illusion of feeling present in the virtual world are more effective at reducing pain (Hoffman et al., 2004c, 2006, 2014; Dahlquist et al., 2007; Wender et al., 2009; Law et al., 2011; Zeroth et al., 2019). Interacting with the virtual world increases the immersiveness of the VR system, potentially increasing the amount of attention drawn into the virtual world (e.g., Wender et al., 2009). The results of these laboratory studies have helped guide the design of effective VR analgesia systems, e.g., fMRI magnet-friendly wide field of view VR helmets, e.g., Hoffman et al. (2004b, 2007) and the development robot-like arm VR goggle holders for severe burn patients (Maani et al., 2008, 2011a,b; Hoffman et al., 2019).

Unfortunately, increasing the immersiveness of VR systems used in the ICU for children with large severe burn injuries is both highly recommended, but also technically challenging. Burns on their heads and face often preclude burn patients from wearing a traditional VR helmet, so head tracking is not possible. Children with large severe burn injuries also often have severe burns on their hands/fingers, making it difficult for them to use mouse tracking to interact with the virtual world during burn wound care.

In the current pilot laboratory study, we used a new VR helmet that allows participants to use their eye movements as a “hands free” input device to interact with the virtual world. We predicted that adding interactivity via an eye tracking system embedded into the VR goggles would increase the immersiveness of the VR system, and would increase the participants illusion of “being there” in the virtual world, making VR more attention grabbing and more effective at reducing the acute pain of healthy volunteers during brief thermal pain stimuli. Several large computer companies are developing and marketing new virtual reality and augmented reality eye tracking technologies. https://www.forbes.com/sites/solrogers/2019/02/05/seven-reasons-why-eye-tracking-will-fundamentally-change-vr/#16bfb2c83459. For example, Apple Computers recently purchased SMI. Note that SMI is the company that made the eye tracking technology used in the current study.

The current analog laboratory pain study with healthy volunteer participants is the first controlled study in the PubMed literature to explore whether interactive eye tracking can enhance the analgesic effectiveness of virtual reality distraction. The SMI eye tracked HTC VIVE VR helmet starts with a standard HTC VIVE helmet. But in addition, each eyepiece of the goggles is trimmed with a small ring of eye tracking technology^2^. Six infrared lights are positioned in a circle around each eye. In addition to the low energy infrared lights, miniature infrared cameras mounted onto the same ring record the pattern of red lights with an infrared camera (see Figure 3). These miniature cameras can make real time digital video streams of the six small red dots of infrared light reflected off of the outer surface of the patient’s eyes (the cornea). As the participants look at different objects in the computer generated world, the pattern of infrared dots changes shape. The VR computer can tell from the pattern of dots, where the patient is looking (search www.youtube.com for “SMI eyetracked virtual reality” for related informational videos). Because the eye tracking system only uses light in the narrow bandwidth of infra-red, the video camera is able to ignore confusing reflection noise from the visible spectrum and infrared thus improves eye tracking accuracy.

The information from the miniature infrared cameras mounted in the VR helmet is transmitted to the VR software program in the VR computer. In the current study, participants interact with the virtual world by aiming virtual snowballs at virtual objects in the 3D virtual canyon. Just as a computer mouse input device can be used to move a computer cursor around a computer screen, using eye tracking technology embedded into the VR goggles, in the current study, the participant in virtual reality can aim snowballs at objects in virtual reality by simply looking at the virtual objects. Essentially, the “cursor” or reticle crosshair, follows the patient’s eye fixations. So if the patient looks at a Snowman in virtual reality, the virtual snowballs hit the Snowman, and the virtual snowman reacts (with special animated effects) when hit by a snowball.

The current laboratory thermal pain study with healthy volunteers explores for the first time, whether interactive eye tracking can enhance the analgesic effectiveness of virtual reality distraction.

## Materials and Methods

### Subjects

Forty-eight female college student volunteers from Effat University (age range 18–30 years old, mean = 21.77, *SD* = 2.07) participated in the main study, and an additional 24 students (from the identical context as the main study participants) were randomized to participate in a small pilot side study, to test our pain paradigm assumption that pain ratings were stable over repeated stimulations for people who received two test pain stimuli with No VR. Effat University is an all female institution of higher education for women in Jeddah Saudi Arabia. All data was collected by female research assistants and all participants were female, an understudied gender. This research was conducted in accordance with the Declaration of the World Medical Association^3^. All subjects gave written informed consent in accordance with the Declaration of Helsinki. Both written and verbal informed consent were obtained using a protocol approved by the Effat University’s Human Subjects Review Committee.

### Within-Subjects Design

Each of the 48 participants who received VR rated their pain during “eye tracked VR” during one thermal pain stimulus (e.g., Test 1), and rated their pain again during a second thermal stimulus during “passive VR” (e.g., Test 2). Treatment order of passive VR vs. interactive eye tracked VR was randomized using random number sequences from www.random.org. Some people received “eye tracked VR” first and “passive VR” second, and some people received “passive VR” first and “eye tracked VR” second. Each individual participant’s pain during passive VR was compared to that same participant’s pain during interactive eye tracked VR.

### Measures and Procedures

#### Experimental Thermal Pain Model

Controlled thermal pain stimulation was applied using a commercially available computerized Medoc thermal pain stimulator^4^ (Medoc Q-Sense Ramp and Hold program). During the first phase of the study, each participant selected the temperature they would use in this study. The stimulus temperature (range = 44 – 48.5°C in the present study) of each 10 s heat stimulus temperature was individually determined for each subject using the psychophysical method of ascending levels (Hoffman et al., 2004b, 2007). A 10-s heat stimulus (always 44°C for the first stimulus) was delivered via a thermode attached to the participant’s forearm (by a female researcher), and the subject was asked to rate their pain during the stimulus using a 0–10 graphic rating scale. With the subject’s permission, the temperature for the next stimulus was then increased by 1°C (or less, if the participant was approaching their maximum) and participants again rated their pain. This sequence was repeated until the subject reported a stimulus that was “painful but tolerable” for the brief stimulus duration, and that the subject was willing to receive for two additional 10-s thermal pain stimuli. This final stimulus temperature that the participant selected for the baseline pain condition (10-s thermal stimulus with no distraction) also served as the pain stimulus temperature used during the subsequent VR interventions (10 s of thermal pain during passive VR distraction, and 10 s of thermal pain during interactive eye-tracked VR, VR treatment order randomized). Allowing participants to select the temperature they would use in this experiment was popular with the participants.

**FIGURE 1:**
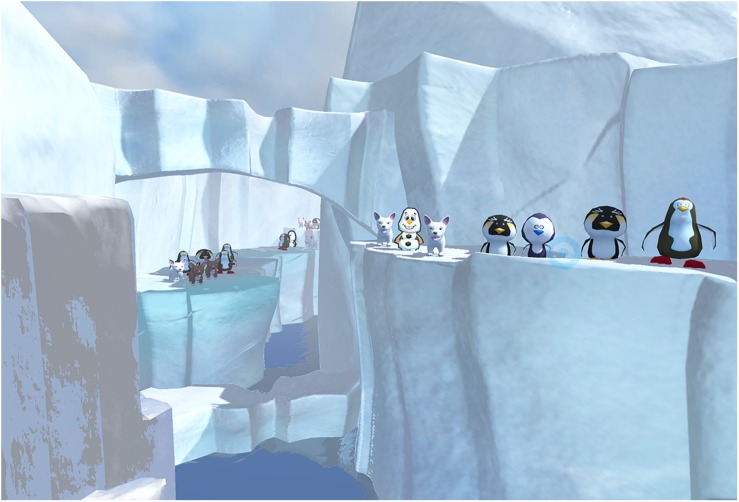
A still shot from SnowCanyon (image by bigenvironments.com, copyright Hunter Hoffman, www.vrpain.com).

**FIGURE 2:**
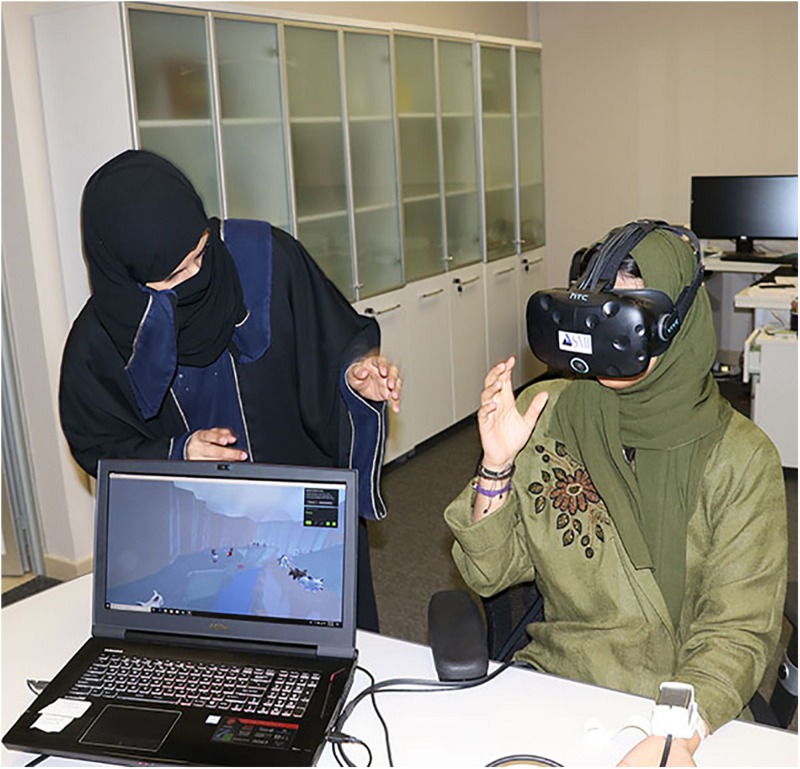
Researcher with a student volunteer participant during the laboratory pain study (photo and copyright Hunter Hoffman, UW, www.vrpain.com).

**FIGURE 3:**
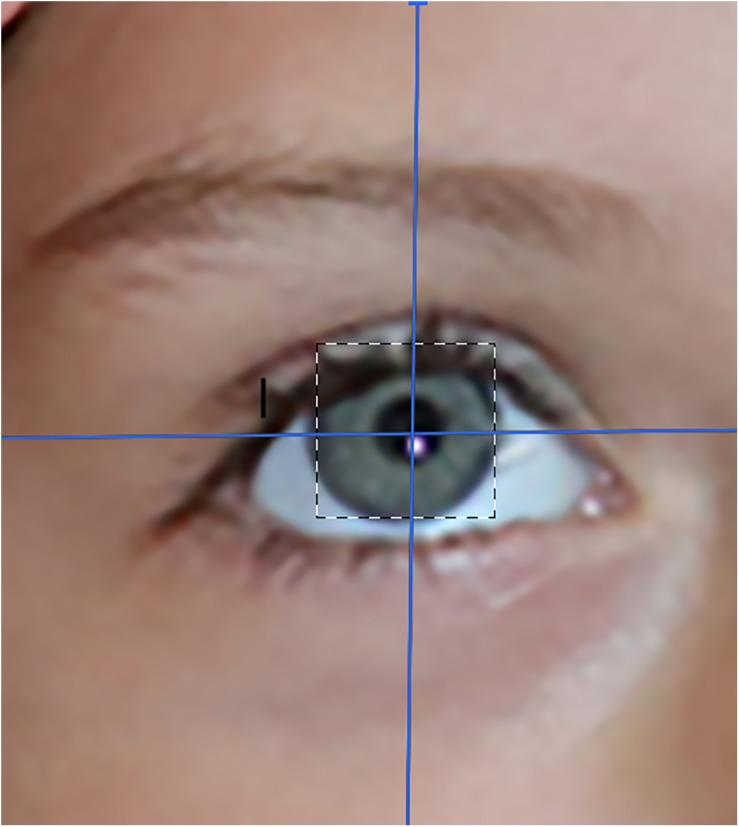
An artist’s rendition of an eye-tracked eye. Photo, image and copyright Hunter Hoffman, www.vrpain.com.

The VR system was carried out using a gaming laptop: MSI GeForce GTX 1080 8 GB, Intel Core i7 7th (2.80 GHz), 16 GB RAM, Windows 10 operating system connected to an SMI HTC VIVE VR helmet with FOV 110° from HTC, with 1080 × 1200 pixels per eye resolution and a refresh rate of 90 Hz. The head mounted display VR helmet, integrated with SMI eye-tracking 250 Hz, works with the SDK C + + \C# for various VR engines like Unity. A new VR world, SnowCanyon^5^^,^^6^ was integrated with the eye tracking hardware, enabling participants to use the eye-tracker to select a virtual object by simply looking at the virtual object target (e.g., a virtual snowman) in the VR goggles. The SnowCanyon virtual environment^5^ presents a virtual arctic canyon to the user, complete with flowing river below, blue sky above, and terraced canyon walls to the sides containing virtual penguins, igloos, and snowmen (see Figure 1). Subjects in both treatment conditions wore an HTC VIVE VR helmet head-mounted display with an integrated SMI eye tracking system (see Figures 2, 3). For all participants in the current study, sound was muted and the helmets were immobilized (no head tracking), as an analog to the robot-like articulated arm goggle held eye-tracked VR goggles that will eventually be used with actual burn patients in future clinical studies. The eye-tracking interactions in the VR game were designed to be easy for the participants to make sense of how the players interact with game objects in that game/environment.

In both VR treatment conditions, each subject “glided” through the virtual world along a pre-determined path. During the interactive VR treatment condition, participants in SnowCanyon could target and shoot virtual objects in the virtual world by moving their eyes (i.e., simply looking at an object to aim snowballs at that object). The Passive VR treatment condition consisted of the identical SnowCanyon software and VR system, but with no eye-tracking and no interactivity/no snowballs. Subjects passively glided through the snowy 3D canyon in the 110 degree field of view HTC VIVE VR goggles.

#### Measures

After each pain stimulus, subjects received the following instructions prior to answering six separate subjective questions: “Please indicate how you felt during the most recent 10-s pain stimulus by making a mark anywhere on the line. Your response does not have to be a whole number.”

After each pain stimulus, participants rated their pain using Graphic Rating Scales (GRS). Such pain rating scales have been shown to be valid through their strong associations with other measures of pain intensity, as well as through their ability to detect treatment effects (Jensen and Karoly, 2001; Jensen, 2003; Hoffman et al., 2004c). The GRS is a 10-unit horizontal line labeled with number and word descriptors. In the current study, the tool was used to assess three reports of the pain experience (“worst pain,” “pain unpleasantness,” and “time spent thinking about pain”) that correspond to three separable components of the pain experience; sensory pain, affective pain, and cognitive pain, respectively.

Descriptor labels were associated with each mark to help the participant rate their pain magnitude in each domain. For pain intensity, the GRS descriptors were *no pain at all, mild pain, moderate pain, severe pain*, and *excruciating pain*. For pain unpleasantness, the GRS descriptors were *not unpleasant at all, mildly unpleasant, moderately unpleasant, severely unpleasant*, and *excruciatingly unpleasant.* For time spent thinking about pain, the GRS descriptors were *none of the time, some of the time, half of the time, most of the time, all of the time.*

The Graphic Rating Scale has previously been used to assess pain intensity in children eight and older and has been documented to be the preferred report method for young children (Tesler et al., 1991). The GRS is more sensitive than simple descriptive pain scales and participants can easily answer these pain ratings despite having no previous experience. Visual Analog Scales have been validated for use in children aged 7 and higher (Bringuier et al., 2009).

A single rating “to what extent did you feel like you ‘went into’ the virtual world,” adapted from Slater et al. (1994) was also used in the present study to assess user presence in the virtual world. Descriptor labels were *I did not feel like I went inside at all, mild sense of going inside, moderate sense of going inside, strong sense of going inside, I went completely inside the computer generated world*. Hendrix and Barfield (1995) showed the reliability of a similar VR presence rating. The measure’s ability to detect treatment effects (Hoffman et al., 2003a, 2004c) is preliminary evidence of our VR presence measure’s validity. Participants also rated how real the objects seemed in virtual reality, descriptors were *completely fake, somewhat real, moderately real, very real, indistinguishable from a real object*. Participants rated nausea as a result of VR, using a graphic rating scale with descriptors *no nausea at all, mild nausea, moderate nausea, severe nausea, vomit.* As a surrogate measure of positive affect, participants rated how much fun they had during the painful stimulus (Hoffman et al., 2004a). The verbal descriptors associated with the fun rating were *no fun at all* (0), *mildly fun* (1–4), *moderately fun* (5–6), *pretty fun* (7–9), and *extremely fun* (10). Previous studies indicate that these secondary measures are sensitive to manipulations of the immersiveness of the VR system (Hoffman et al., 2004a, c; Wender et al., 2009).

The specific questions used in the current study were designed to assess the cognitive component of pain (amount of time spent thinking about pain), the affective component of pain (pain unpleasantness), and the sensory component of pain (worst pain). Nausea/Dizziness was assessed in an effort to identify the incidence of this component of simulator sickness sometimes associated with VR use.

Participants individually identified and pre-approved a baseline thermal pain stimulus temperature they found “painful but tolerable for 10 s, that they were willing to tolerate for two more 10 s thermal pain stimuli at that same temperature.” The mean thermal stimulation temperature was 47.18°C (*SD* = 0.93).

Subjective pain ratings were obtained from each healthy volunteer participant after brief thermal pain stimuli at three time points, with an interstimulus interval of approximately 4 min, using the same temperature each time: (a) baseline, (b) Test 1, and (c) Test 2.

## Results

As mentioned earlier, treatment order was randomized, using random number sequences from random.org. Twenty-four participants received passive VR first and interactive eye tracked VR second (treatment order 1) and the other 24 participants received interactive eye tracked VR first and passive VR second (treatment order 2).

A two way Mixed ANOVA was conducted to test for undesired treatment order effects. The repeated measures ANOVA factor was (passive VR vs. interactive eye tracked VR), and the between groups factor was treatment order 1 vs. treatment order 2. Mixed ANOVAs showed there was no significant interaction between treatment order and worst pain ratings (i.e., no significant treatment order effects for Worst pain), *F*(1,46) = 1.81, *p* = 0.19 ns, MS = 2.04, ${\text{η}}_{\text{p}}^{2}$ = 0.04. There was no significant interaction between treatment order and pain unpleasantness, *F*(1,46) < 1, *p* = 0.69 ns, MS = 0.26, ${\text{η}}_{\text{p}}^{2}$ = 0.004. There was no significant interaction between treatment order and participants ratings of Time spent thinking about pain during the thermal stimulus, *F*(1,46) = 1.00, *p* = 0.32 ns, MS = 0.51, ${\text{η}}_{\text{p}}^{2}$ = 0.02. And, there was no significant interaction between treatment order and participants ratings of Fun during the thermal stimulus, *F*(1,46) = 1.45, *p* = 0.23 ns, MS = 1.76, ${\text{η}}_{\text{p}}^{2}$ = 0.03.

Because no significant order effects were found, the results were collapsed across treatment order for all of the analyses below.

### Statistical Analyses Collapsed Across Treatment Order

One way repeated measure ANOVAs were performed to test if there were significant main effects of No VR vs. passive VR vs. interactive eye-tracked VR. Paired *t*-test analyses were performed for the primary outcome measure (worst pain), as well as for the secondary pain measures (i.e., pain unpleasantness, and time spent thinking about pain). For these three pain ratings, alpha was conservatively set at 0.05/3 = 0.017. Any *p*-value less than 0.017 was considered significant (Bonferroni corrected for familywise error). Additional paired *t*-test analyses were performed for the other secondary graphic rating scale measures (fun, nausea, presence, real) with α = 0.05.

#### WORST PAIN

A one way repeated measure ANOVA indicated a significant main effect of No VR vs. passive VR vs. interactive eye-tracked VR for worst pain, *F*(2,94) = 61.41, *p* < 0.001, MS = 92.15, partial η^2^ = 0.57. As predicted, compared to No VR, worst pain ratings were significantly lower during passive VR. Compared to No VR, worst pain was significantly lower during interactive eye tracked VR. And compared to passive VR, worst pain was significantly lower during interactive eye tracked VR.

**Table:** 

**Worst pain**

**No VR**	**Passive VR**	**Eye tracked VR**	**Paired *t*-tests**

6.35 (1.14)	5.00 (1.91)		*t*(47) = 5.66, *p* < 0.001, *SD* = 1.66
6.35 (1.14)		3.58 (2.03)	*t*(47) = 9.64, *p* < 0.001, *SD* = 1.99
	5.00 (1.91)	3.58 (2.03)	*t*(47) = 6.49, *p* < 0.001, *SD* = 1.51

### Pain Unpleasantness

A one way repeated measure ANOVA showed a significant main effect of No VR vs. passive VR vs. interactive eye tracked VR for pain unpleasantness, *F*(2,94) = 44.33, *p* < 0.001, MS = 68.92, ${\text{η}}_{\text{p}}^{2}$ = 0.49.

*Post hoc* paired comparisons (paired *t*-tests) are shown below. As predicted, compared to No VR, pain unpleasantness ratings were significantly lower during passive VR. Compared to No VR, pain unpleasantness was significantly lower during interactive eye tracked VR. And compared to passive VR, pain unpleasantness was significantly lower during interactive eye tracked VR.

#### PAIN UNPLEASANTNESS

**Table:** 

**Pain unpleasantness**			
**No VR**	**Passive VR**	**Eye tracked VR**	**Paired *t*-tests**
4.73 (1.62)	3.48 (2.18)		*t*(47) = 5.15, *p* < 0.001, *SD* = 1.68
4.73 (1.62)		2.33 (1.86)	*t*(47) = 8.96, *p* < 0.001, *SD* = 1.85
	3.48 (2.18)	2.33 (1.86)	*t*(47) = 4.53, *p* < 0.001, *SD* = 1.75

### Time Spent Thinking About Pain

A one way repeated measure ANOVA (using Greenhouse–Geisser) showed a significant main effect of No VR vs. passive VR vs. interactive eye tracked VR for “time spent thinking about pain,” *F*(2,94) = 28.83, *p* < 0.001, MS = 79.49, ${\text{η}}_{\text{p}}^{2}$ = 0.38.

*Post hoc* paired comparisons (paired *t*-tests) are shown below. As predicted, compared to No VR, participants ratings of time spent thinking about pain during the thermal stimulus were significantly lower during passive VR. Compared to No VR, time spent thinking about pain was significantly lower during interactive eye tracked VR. But contrary to predictions, compared to passive VR, time spent thinking about pain was NOT significantly lower during interactive eye tracked VR.

#### TIME Spent Thinking About PAIN

**Table:** 

**Time spent thinking about Pain**			
			***Post hoc***
**No VR**	**Passive VR**	**Eye tracked VR**	**paired *t*-tests**
2.35 (2.40)	0.625 (1.27)		*t*(47) = 5.54, *p* < 0.001, *SD* = 2.16
2.35 (2.40)		0.479 (0.99)	*t*(47) = 5.71, *p* < 0.001, *SD* = 2.28
	0.625 (1.27)	0.479 (0.99)	*t*(47) = 1.00, *p* = 0.32 NS, *SD* = 1.01

### Fun During the Thermal Stimulus

A one way repeated measure ANOVA showed a significant main effect of No VR vs. passive VR vs. interactive eye tracked VR for “fun,” *F*(2,94) = 107.40, *p* < 0.001, MS = 234.65, ${\text{η}}_{\text{p}}^{2}$ = 0.70.

*Post hoc* paired comparisons (paired *t*-tests) are shown below. As predicted, compared to No VR, participants’ ratings of fun during the thermal stimulus were significantly higher during passive VR. Compared to No VR, fun was significantly higher during interactive eye tracked VR. And compared to passive VR, fun was significantly higher during interactive eye tracked VR.

#### FUN

**Table:** 

**Fun**			
**No VR**			***Post hoc***
**(baseline)**	**Passive VR**	**Eye tracked VR**	**paired *t*-tests**
1.40 (2.11)	3.79 (2.21)		*t*(47) = 7.38, *p* < 0.001, *SD* = 2.25
1.40 (2.11)		5.81 (2.42)	*t*(47) = 12.92, *p* < 0.001, *SD* = 2.37
	3.79 (2.21)	5.81 (2.42)	*t*(47) = 8.95, *p* < 0.001, *SD* = 1.56

#### NAUSEA FROM VIRTUAL REALITY

No significant difference in “nausea during VR” was found between passive VR and interactive eye tracked VR.

**Table:** 

**Nausea**			
			***Post hoc***
**No VR**	**Passive VR**	**Eye tracked VR**	**paired t-tests**
	0.146 (0.51)	0.21 (0.92)	*t*(47) < 1. NS, *p* = 0.685, *SD* = 1.06

### Presence

Compared to their illusion of presence during passive VR, participants reported having a significantly stronger illusion of presence in virtual reality (being there), during interactive eye tracked VR.

#### PRESENCE IN VIRTUAL REALITY

**Table:** 

**Presence**			
			***Post hoc***
**No VR**	**Passive VR**	**Eye tracked VR**	**paired t-tests**
	4.31 (2.23)	6.14 (2.65)	*t*(47) = 6.24, *p* < 0.001, *SD* = 2.04

### Real

Participants rated the virtual objects as significantly more real during interactive eye tracked VR, compared to during passive VR.

#### HOW REAL WERE THE OBJECTS IN VIRTUAL REALITY

**Table:** 

**Real**			
			***Post hoc***
**No VR**	**Passive VR**	**Eye tracked VR**	**paired *t*-tests**
	3.75 (2.14)	4.75 (2.47)	*t*(47) = 5.12, *p* < 0.001, *SD* = 1.35

In order to test an important assumption of our thermal pain paradigm, pilot data collected from an additional 24 participants in the same context received No VR during baseline, No VR during Test 1 vs. No VR again during Test 2 (see Table 1). As predicted, participants who received No VR did not habituate to the thermal pain stimuli. In other words, the pain ratings from the thermal pain stimulations were stable over repeated pain stimulations for people who received one baseline pain and two test pain stimuli with No VR, using the same thermal pain paradigm as the main study. As predicted, as shown in Table 1, three separate Bonferroni corrected One-Way repeated measure ANOVAs indicated no significant main effect of No VR vs. passive VR vs. interactive eye-tracked VR for worst pain, pain unpleasantness, or time spent thinking about pain.

**TABLE 1:** Results for the control group: one-way repeated measures ANOVAs for worst pain, pain unpleasantness, and time spent thinking about pain.

**Worst pain ratings for the Control Group (*n* = 24 subjects)**			
**Worst pain**			**Mauchly’s *p* = 0.003, sphericity not assumed, Greenhouse–Geiser used. Bonferroni corrected alpha = 0.017, *p* < than 0.017 are significant**
**No VR**	**Passive VR**	**Eye tracked VR**	

6.25 (1.15)	6.38 (1.44)	6.75 (1.48)	*F*(1.41,46) = 4.85, *p* = 0.024, not significant, MS = 2.31, ${\text{η}}_{\text{p}}^{2}$ = 0.17.

**PAIN UNPLEASANTNESS for the Control Group (*n* = 24 subjects)**			
**Pain unpleasantness**			**Mauchly’s *p* = 0.001, sphericity not assumed, Greenhouse–Geiser used. Bonferroni corrected α = 0.017, any *p* < than 0.017 are significant**

**No VR**	**Passive VR**	**Eye tracked VR**	

4.33 (2.26)	4.29 (2.05)	4.83 (2.04)	*F*(1.35,46) = 4.02, *p* = 0.043, not significant, MS = 3.23, ${\text{η}}_{\text{p}}^{2}$ = 0.15

**TIME spent thinking about PAIN for the Control Group (*n* = 24)**			
**Time spent thinking about Pain**			**Mauchly’s *p* = 0.002, sphericity not assumed, Greenhouse–Geiser used. Bonferroni corrected α = 0.017, any *p* < than 0.017 are significant**

**No VR**	**Passive VR**	**Eye tracked VR**	

2.96 (2.68)	2.17 (2.71)	2.75 (3.03)	*F*(1.41,46) = 5.08, *p* = 0.021, not significant, MS = 5.75, ${\text{η}}_{\text{p}}^{2}$ = 0.18

## Discussion

In the current study, we measured the brief acute pain of healthy volunteers during 10 s thermal pain stimuli to test whether increasing the immersiveness of the VR system increased how effectively VR reduces acute pain during brief thermal stimulations. As predicted, compared to passive VR, interactive eye tracked VR was significantly more effective at reducing worst pain (sensory pain), more effective at reducing pain unpleasantness (the emotional component of pain), and interactive eye tracked VR was more fun than passive VR. Furthermore, as predicted, compared to the passive VR condition, participants rated their illusion of presence significantly higher during the interactive eye tracked VR condition, and virtual objects seemed significantly more real during interactive VR, compared to passive VR.

In addition, the current study also tested an important assumption of our thermal pain paradigm. Pilot data collected from an additional 24 participants in the same context, received No VR during baseline, No VR during Test 1, and No VR again during Test 2. As predicted, the 24 participants who received No VR reported no reduction in pain. In other words, the pain ratings from the thermal pain stimulations were stable over repeated pain stimulations for people who received one baseline pain and two test pain stimuli with No VR, using the same thermal pain paradigm as the main study.

### Limitations

The within-subjects study design used in the current study reduces noise variance and increases statistical power. However, one limitation of our current study is that researchers and subjects were not blinded to the treatment conditions (Campbell and Stanley, 1963; Schulz and Grimes, 2002). In the current study, VR was “visual only” with no sound effects. Having sounds muted may have exaggerated the benefit from the eye movement interactivity. One of the advantages of VR is the multimedia exposure. Sounds of rivers running and birds singing enhance the illusion of presence and heighten the participant’s sensitivity to the 3D environment. On the other hand, the lack of sound effects in the current study may have underestimated the benefits of eye movement interactivity, because there would be more sound effects in the interactive VR condition, which could make interactive VR more distracting and make the advantage of interactive VR over passive VR even more pronounced. Another limitation is that this study did not investigate/measure participants’ attention level (e.g., Heathcote et al., 2017). In the current study, eye tracking technology was simply used as a naturalistic human computer interface. Future, more advanced versions of our new VR analgesia system may use eye blinks and duration of fixation to help define interactions, and further increase interactivity. In the current study, “hands free” eye tracked interactive VR was compared to passive non-interactive VR. Whether eye tracking increases analgesia compared to other “hands free” interactive VR systems (e.g., voice controlled, etc.) remains a possible topic for future research. In the current study, each individual selected a temperature they found “painful but tolerable for 10 s.” Clinical research is needed to determine whether the current results generalize to clinical pain settings (e.g., for 20 min wound cleaning procedures of severe burn patients that often involve severe to excruciating pain). But, encouragingly, our first pediatric burn patient pilot subject, using the same eye tracked VR analgesia system, reported unusually large reductions in pain during burn wound care in the intensive care unit, during eye-tracked VR vs. No VR.

Despite these limitations, the current study is the first PubMed indexed VR analgesia study to involve eye tracking technology embedded into the VR goggles. Although there is a large scientific literature on traditional eye tracking spanning more than 40 years of research (Kredel et al., 2017), there are very few studies combining eye tracking and immersive virtual reality, a very recent innovation/combination.

### Why Does VR Reduce Pain? Possible Mechanisms of VR Analgesia

Although there is growing evidence that VR can be effective for reducing acute pain during painful medical procedures (Hoffman et al., 2019), the non-pharmacologic mechanism of how VR reduces pain is not fully understood and is an important research topic. According to Ballantyne (2018, p. s25) “The conscious perception of pain depends on the conversion of nociception to perception…”. The authors of the current study speculate that VR interferes with the conversion of nociception to conscious pain perception by inserting a powerful perceptual illusion into the painful experience. Instead of directing most of their attention toward converting nociceptive signals into pain perception during No VR, we speculate that during virtual reality, the patient’s brain is pre-occupied with converting neural signals from the visual, auditory and other sensory systems into a multisensory perceptual illusion of “presence” in virtual reality.

In the current study, we predicted that adding hands free interactive eye tracking would make VR that much more attention grabbing, and thus more effective at reducing pain, compared to passive VR. As predicted, participants in the current study also reported a significantly stronger illusion of presence, and VR was significantly more fun during the eye tracked VR. Although the current study did not directly measure attention, the pattern of results of the current study are consistent with an attentional mechanism of how VR reduces pain (see also Hoffman, 1998, 2004; Gold et al., 2006, 2007; Dahlquist et al., 2007; Birnie et al., 2017; Gold and Mahrer, 2017; Zeroth et al., 2019).

### Future Directions

Whether the current results generalize to clinical patient populations is an important topic for future research (e.g., whether eye tracking increases VR analgesia effectiveness for pediatric burn patients during burn wound care). In the current laboratory thermal pain study with healthy volunteers, eye movements were used to tell the computer what virtual objects the participant was looking at in virtual reality. In future studies on VR analgesia (e.g., virtual reality pain distraction), eye tracking technology can also be used to collect data about the patient’s current mental state. For example, pupil size, and patterns of eye movements may correlate with how much pain patients are consciously experiencing. When a burn patient’s pain becomes so extreme that the patient’s attention shifts away from VR and onto their pain, we predict large reductions in successful eye fixations on target objects in SnowCanyon.

Immersive Virtual Reality with eye tracking has wide potential clinical uses beyond pediatric burn patients. For example, VR has recently been used with spinal injury patients (Flores et al., 2018). Most paralyzed patients are able to move their eyes, and can thus use eye movements to interact with objects in the virtual world. In the future, eye-tracked virtual reality and also augmented reality glasses may allow people to quantify and improve the efficiency of information processing/learning, etc. And there is also growing interest in using eye tracking to help improve social skills (e.g., helping autistic patients learn to make more natural patterns of eye contact with other humans). Additional research and development is recommended.

## Data Availability Statement

The datasets generated for this study are available on request to the corresponding author.

## Ethics Statement

All subjects gave written informed consent in accordance with the Declaration of Helsinki. Both written and verbal informed consent were obtained using a protocol approved by the Effat University’s Human Subjects Review Committee.

## Author Contributions

All authors listed have made a substantial, direct and intellectual contribution to the work, and approved it for publication.

## Conflict of Interest

After the current study was completed, HH joined the Scientific Advisory Board of BehaVR.com, but no products or funding from this source were involved in the current study. All authors declare that the research was conducted in the absence of any commercial or financial relationships that could be construed as a potential conflict of interest.

## Footnotes

1 https://www.nationalgeographic.com/magazine/2020/01/scientists-are-unraveling-the-mysteries-of-pain-feature/

2 https://en.wikipedia.org/wiki/SensoMotoric_Instruments

3 www.wma.net

4 www.medoc-web.com

5 bigenvironments.com

6 www.vrpain.com
